# Ten simple rules for drawing scientific comics

**DOI:** 10.1371/journal.pcbi.1005845

**Published:** 2018-01-04

**Authors:** Jason E. McDermott, Matthew Partridge, Yana Bromberg

**Affiliations:** 1 Computational Biology and Bioinformatics, Pacific Northwest National Laboratory, Richland, Washington, United States of America; 2 Department of Molecular Microbiology and Immunology, Oregon Health & Sciences University, Portland, Oregon, United States of America; 3 Engineering Photonics, Cranfield University, Cranfield, Bedfordshire, United Kingdom; 4 Department of Biochemistry and Microbiology, Rutgers University, New Brunswick, New Jersey, United States of America; 5 Institute for Advanced Study, Technische Universität München, Garching, Germany; Dassault Systemes BIOVIA, UNITED STATES

Institutions around the world are in a constant struggle to improve science communication. From calls for journal papers to be simpler and more accessible to encouraging scientists to take a more active role through community engagement, there is a drive to demystify and improve public understanding of and engagement with science [1–3]. This drive for engagement is crucial to both helping recruit the next generation of scientist and highlighting the impact and role science has in public life. It also has a role in peer-to-peer communication and wider dissemination of ideas throughout the community. Technology has greatly helped expand the range of teaching styles that a lecturer can call on to reach more people in new ways. Social media outlets like Twitter, Facebook, Instagram, and Tumblr have expanded the reach of science communication within and across scientific disciplines and to the lay public [1, 3]. These new communication channels seem to support endless innovations in the development of videos, interactive quizzes, and instant feedback. Yet they are also providing a platform for a renaissance of one of the simplest and most effective methods for communicating ideas—comics. There are few scientists who haven’t heard of Randall Munroe, the artist behind the web comic “xkcd” [4], which features amazing graphic explanations on everything from climate change [5] to data storage [6]. These comics are widely appealing to a diverse audience and are posted on walls in laboratories and pubs alike. The ideas that they explain are complicated, but by simplifying them down to the core messages and by providing simple visual analogies, the comics educate and engage the groups that other media cannot always reach.

A comic is generally an illustration that employs metaphor and/or storytelling to clearly communicate an idea to a broad audience. Comics often employ humor, but their narratives can be exclusively informational in nature or can deal with nonhumorous topics. Comics can take multiple forms, from the single panel one-liner, to multiple panels, to graphic novels that span multiple pages. There are a number of science- and academic-oriented comics in circulation, including xkcd, PHD [7], and the authors’ own Errant Science [8] and RedPen/BlackPen [9].

An effective comic can communicate difficult ideas efficiently, illuminate obscure concepts, and create a metaphor that can be much more memorable than a straightforward description of the concept itself. Comics can be used to punctuate presentations or journal publications [10–12] to increase impact. In public health education, comics have long been recognized as an effective tool for reaching lots of different populations for education on subjects like cancer [13], fitness [14], and diabetes [15], to name only a few. A recent trend is for scientists and artists (and scientist-artists) to capture the content of talks at conferences, or indeed entire meetings [16], as graphical notes [17]. A vibrant and growing scientific community on social media makes this a particularly effective method for expanding the intended audience; i.e., particularly engaging comics are “virally” spread within very short time frames. Science comics have also been included in research studies to enhance the story and facilitate understanding by a broader audience [10–12]. Certain journals have a “cartoon” category for submission so that the comic will appear in a citable form in publication [18]. Broadly, all of these avenues represent different ways of promoting work to others.

Here, we focus on three key opportunities provided by comics. First, presenting ideas visually is an effective entry point to complex ideas. Second, using metaphor makes information memorable in ways that literal descriptions do not. Third, though not all topics and situations are suited to the use of humor, employing humor can engage nonexperts and experts alike. It both reduces the levels of intimidation associated with presenting scientific results to a wide audience and breaks down the barriers to understanding that often come with new science.

Here, we set out several guidelines that we hope will convince more scientists that drawing your own comics is simpler than you think. We start with breaking the biggest deterrent of all.

## Rule 1: You don’t have to be good at art

**Fig 1:**
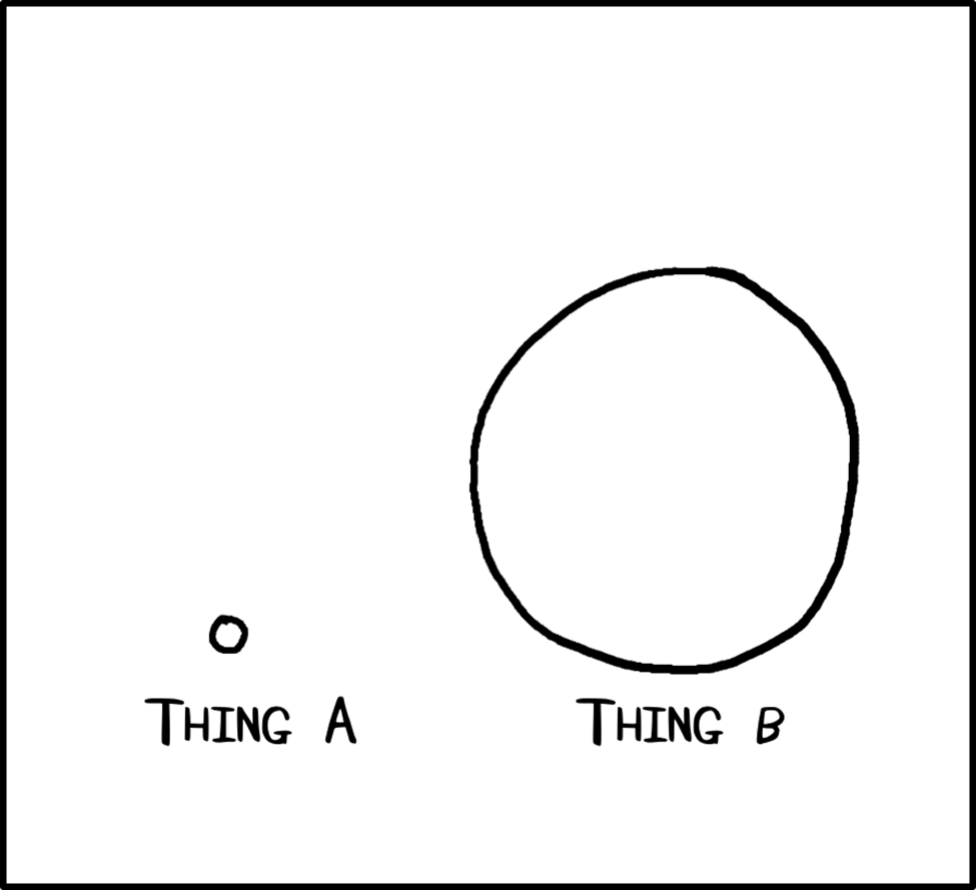
Sense of relative scale can be conveyed with very simple drawings.

**Fig 2:**
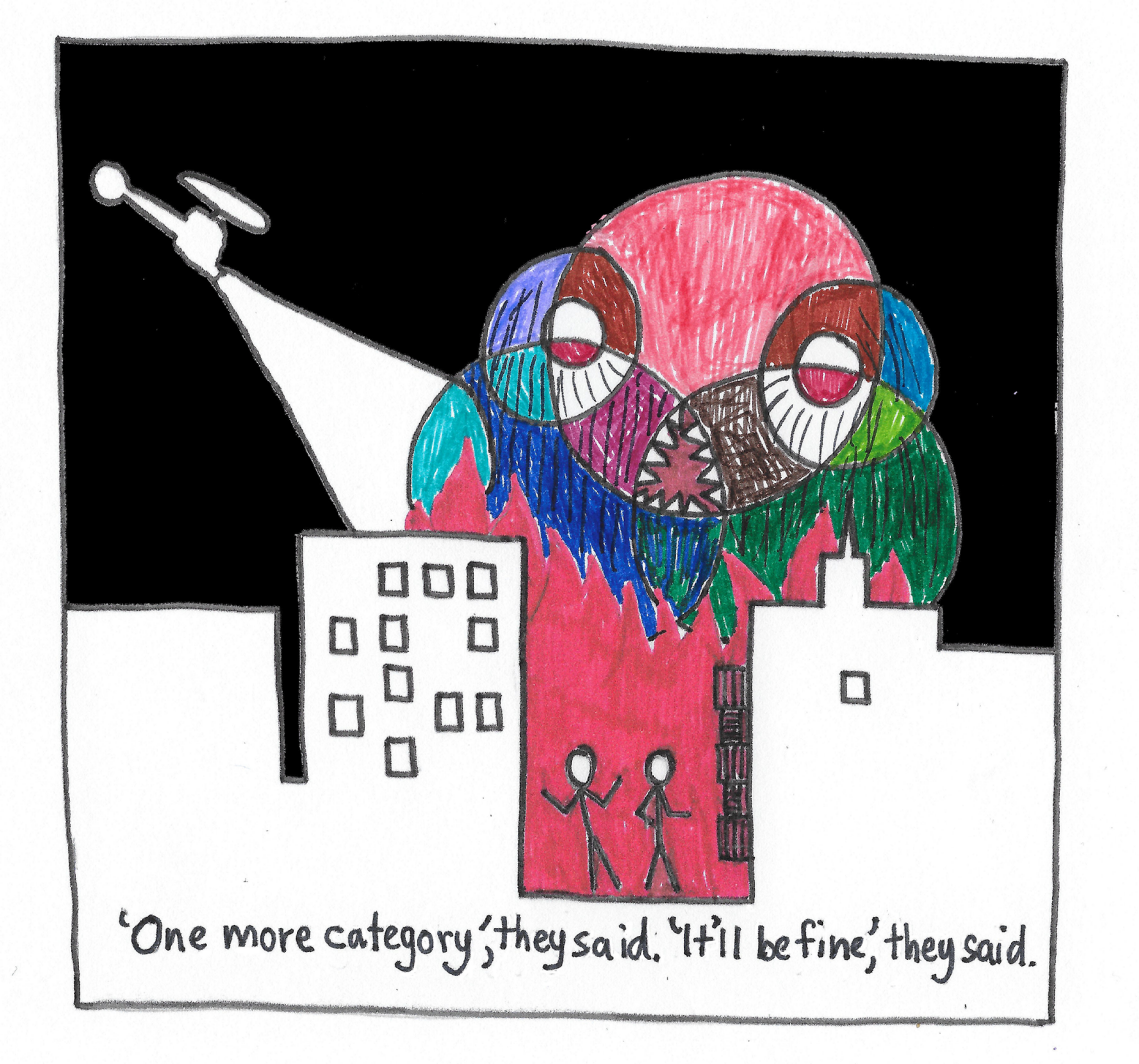
Adding information can create a “Vennster” (the intersection of a Venn diagram and monster).

**Fig 3:**
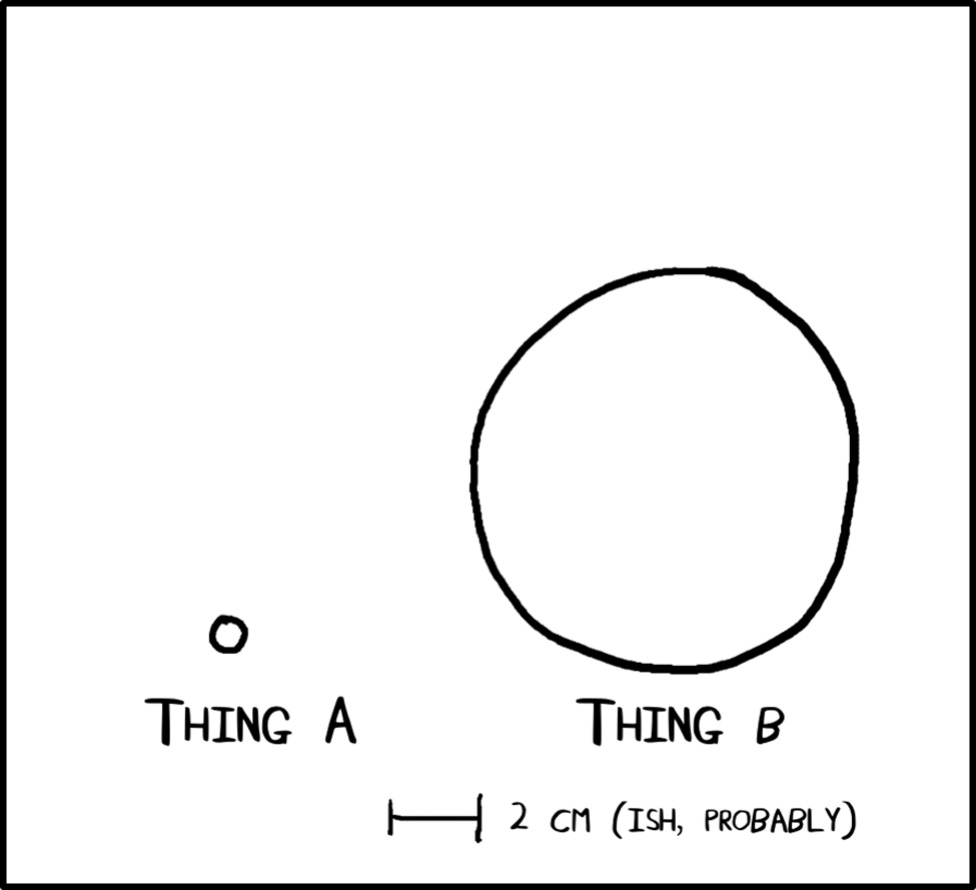
In general use, only enough information to get the idea across.

**Fig 4:**
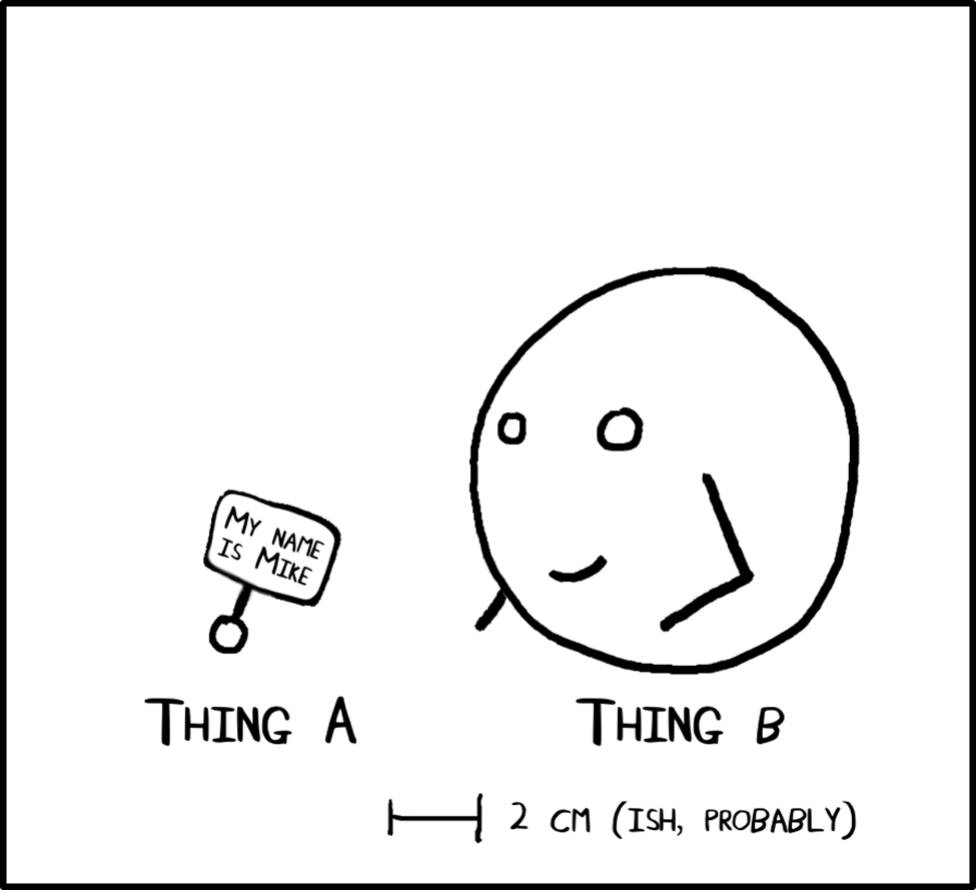
Adding faces and names increases engagement.

**Fig 5:**
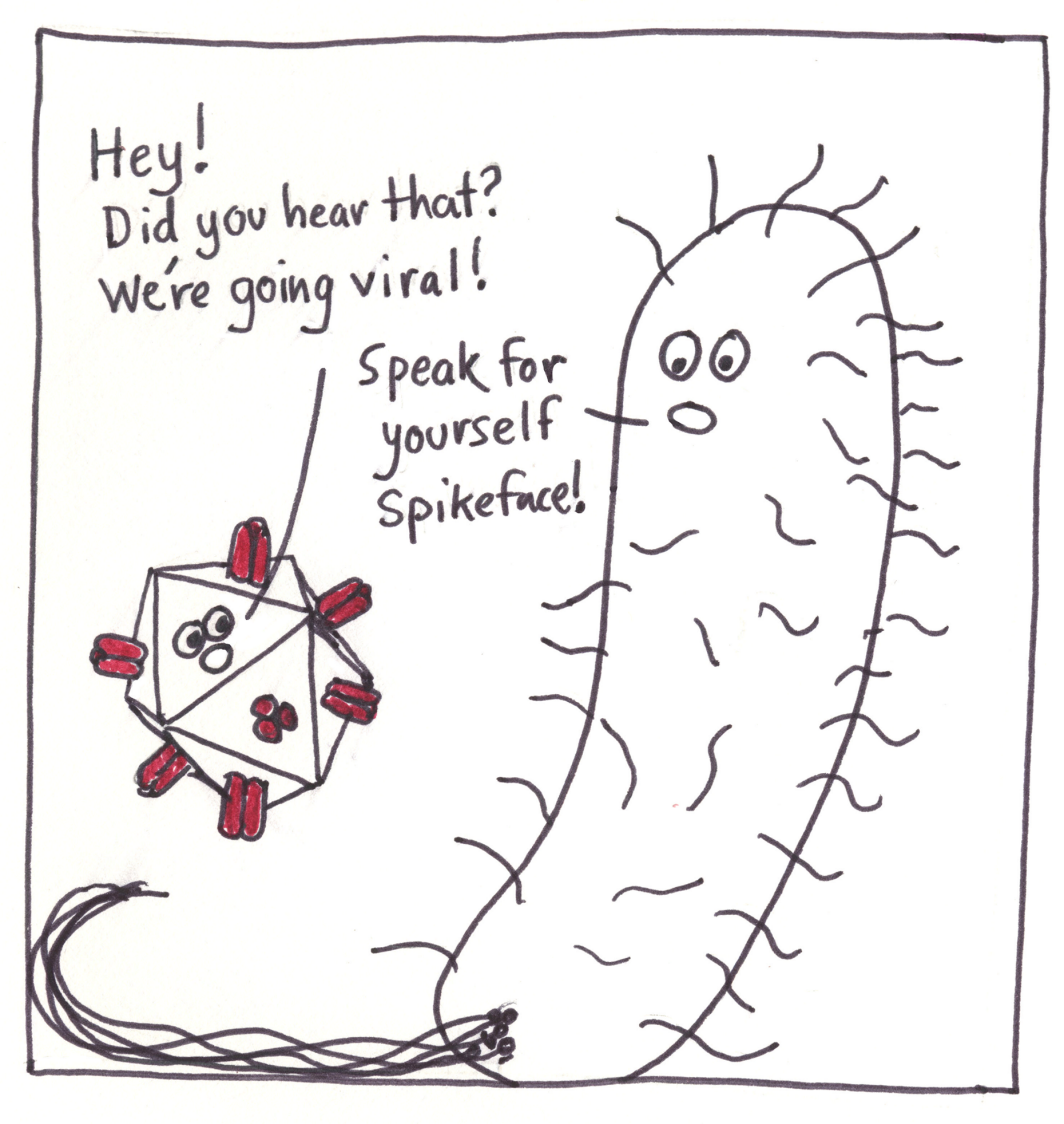
Comics have a way of going viral.

**Fig 6:**
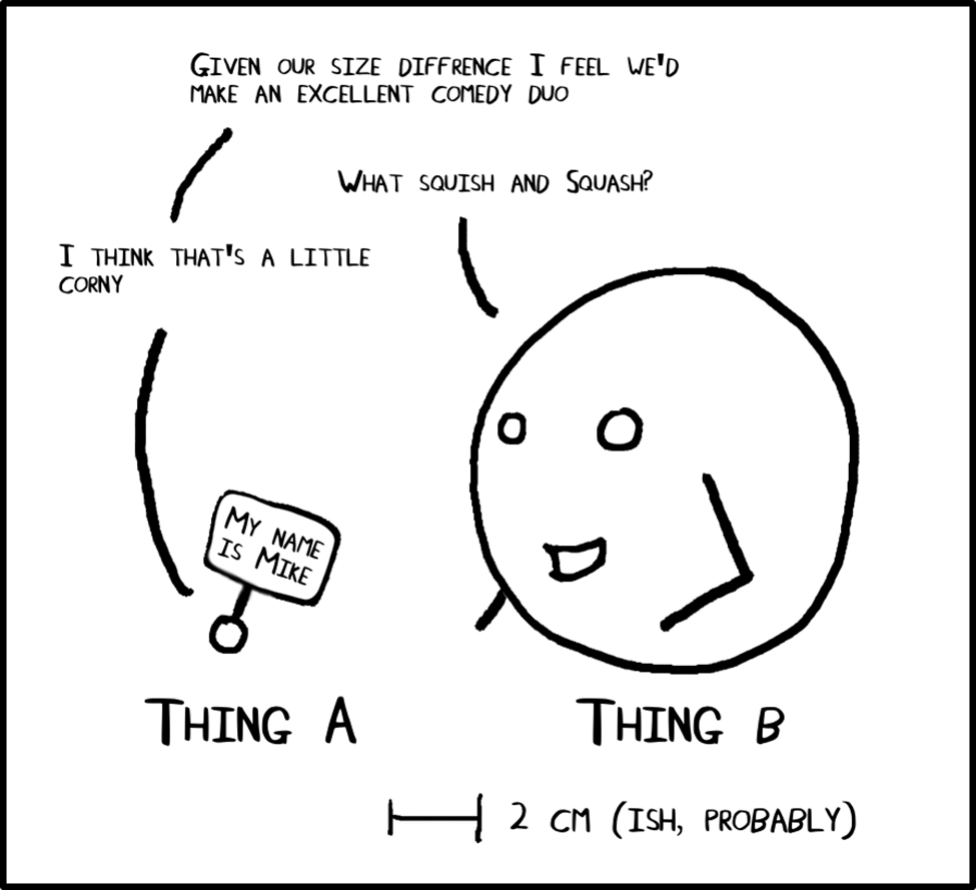
Interaction between characters is an essential part of storytelling.

**Fig 7:**
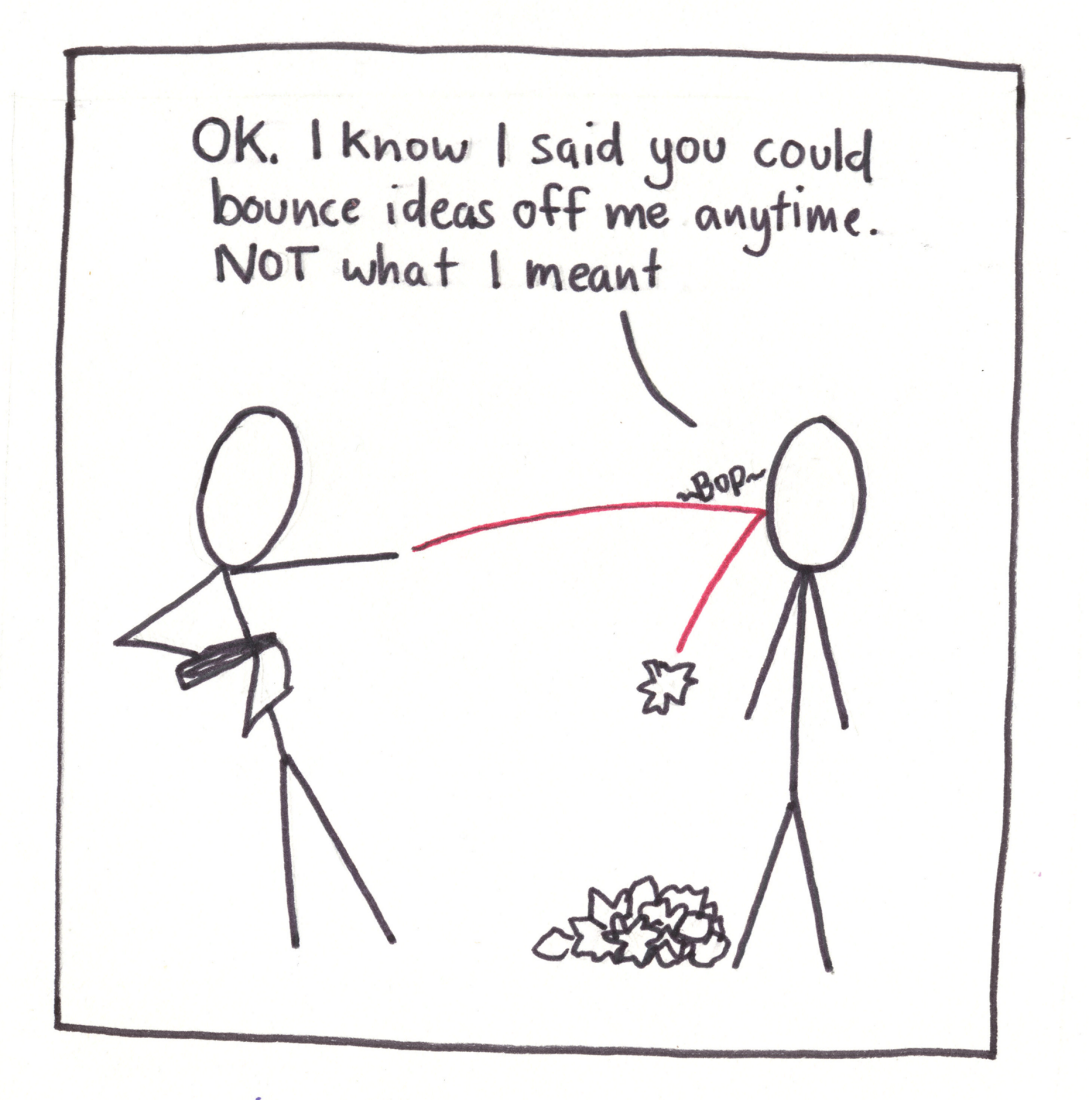
Find a trusted friend to bounce ideas off of.

**Fig 8:**
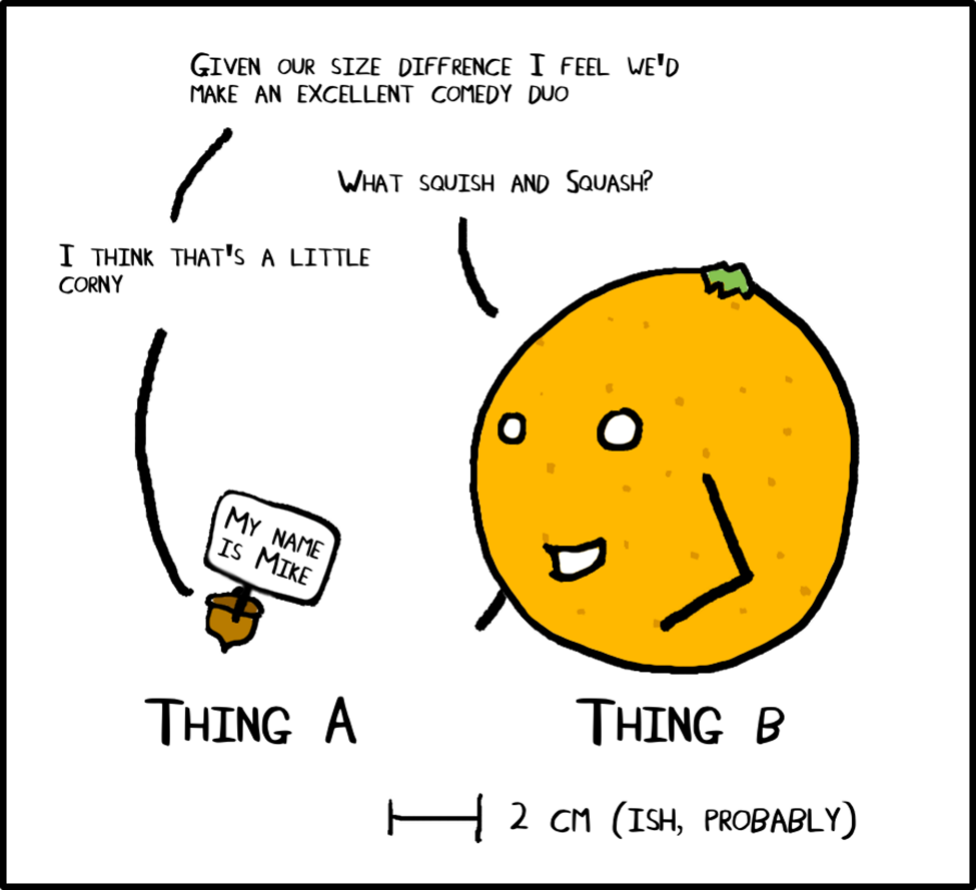
Adding an analogy can transform a comic.

**Fig 9:**
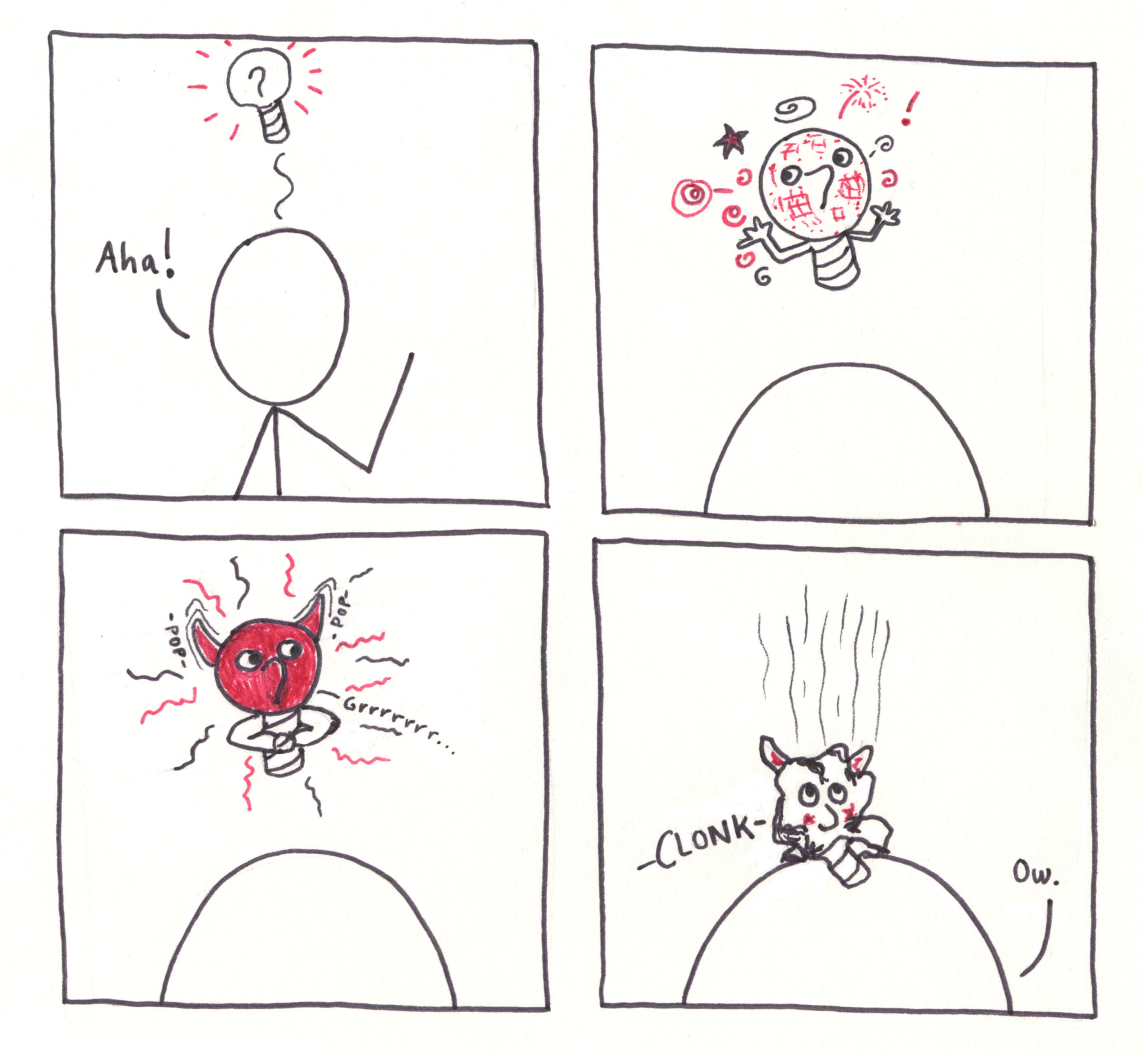
Some ideas take time to develop, others are better fresh.

**Fig 10:**
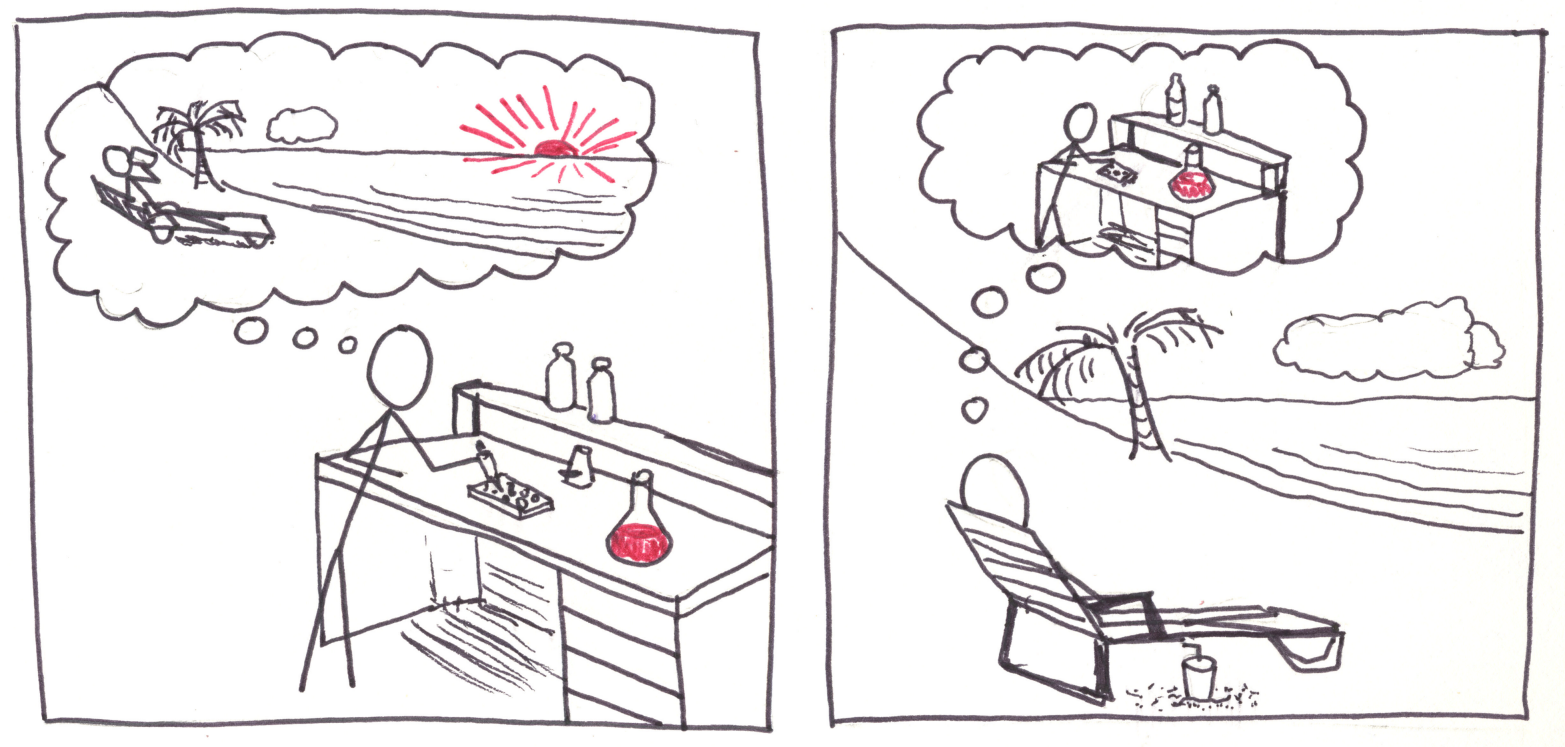
Relax and have fun—in whatever way you can.

Comics are not about art. They are about conveying a message in graphic form. Graphs and plots are for accurately conveying data, diagrams are for accurately depicting a system or setup, and comics are there to help people understand an idea. Some of the best cartoonists and comic artists cannot draw much better than wobbly lines forming strange shapes (Figs 1–10). The trick is to find the shapes that best convey the point you are trying to make. For example, you can convey the sense of scale within a system with a single circle and a dot. Use the dot to represent your smallest scale and then draw a proportionally scaled circle to represent the larger scale. This very basic comic conveys a sense of scale better than writing “small” and “twenty times bigger” (Fig 1). As is explored further, it’s not about the smoothness of the lines or the accuracy of the circles, and if you can make a crude shape on paper, you can do what we set out in these rules. Anyone can create a comic, and often the biggest barrier is just getting over the idea that you can’t. With practice, you’ll get better at communicating ideas this way.

While a piece of paper and a pencil are enough to get started drawing, there are also numerous websites that provide comic drawing software free [19] as well as guides on some of the finer details behind producing full comics [20].

## Rule 2: Comics should be simple

The use of comics should make a complicated idea simpler and easier to understand—not harder! Figure out which of your components or steps can be removed or combined in your comic. Comics are like figures in papers; they are best when each conveys one message. Complicated multithreaded comics can look like a “ridiculogram"—a graph with six axes or a Venn diagram with six categories, one of them shaped like a banana (see Fig 4 from [21]). These are graphical strategies that are fun to look at but cannot be easily interpreted (Fig 2). As with the previous example, the comic works best when conveying a simple message, in that case indicating the scale of the system.

## Rule 3: Make it right, not perfect

Check the science. If your comic has scientific ideas in it, take the time to make sure you have the details right. If it’s mainly just a funny-joke comic, it doesn’t need to be absolutely right. For example, you can add footnotes to comics to point out scientific inaccuracies. But if it’s a comic that is meant to really illustrate a scientific concept for the purpose of education, then it should be as factually correct as you can make it. Including incorrect information in something that is intended to educate is misleading and can lead to misconceptions for those you are trying to reach who may not have a scientific background. In the example of the dot and the circle, no one is going to run a volume analysis on your comic (Fig 3). But they will expect it to be within a by-eye–visible order of magnitude of what you are trying to convey.

## Rule 4: Characters can improve engagement

Create characters with personality that can guide the reader—what your character wears, how tall they are, what they are carrying. If your subjects are inanimate objects, then add personality by including a face. Humans see a face and easily recognize humanity in objects. The famous example is when you hold a pencil, tell everyone that you have named it Steve and then immediately break it [22]. People will tend to feel empathy for the pencil. Simply naming your shapes can be enough to help people engage with the comic and understand and remember the message it conveys. Personification allows the expression of emotions and interactions between players in your comic that let a story be told (see Rule 6). In the dot and circle example, this can be as simple as giving one of the objects hand-like shapes (Fig 4). Or in a more real-world setting, adding something as simple as googly eyes to equipment can produce the same result.

## Rule 5: Don’t punch down

Comics have a way of going viral (Fig 5), and it’s a good idea to reflect on the possible consequences of everyone in the world reading your comic. (No, not literally everyone in the world.) Don’t punch down: making mean fun of those less powerful or privileged than you is bad form, and you should evaluate with every comic you produce. Maintaining a spirit of fun, self-effacing humor and/or commiseration can often express similar ideas without putting anyone down. Be careful with work-inspired comic ideas. Complaining about your workplace using specific details is simply not a good idea. If you do, try not to make any situation or anyone in the comic identifiable—unless you’ve asked them first or they’re a public figure. It shouldn’t need to be said, but avoid jokes that are sexist, racist, ableist, or most other “ists.” (Marxist jokes may be back on the table.) You should really avoid those in real life as well. If you do get criticized for a comic you’ve posted, take a deep breath, let it out, find a trusted and honest friend or colleague, and ask their opinion. Don’t be afraid to pull the comic. There are rare cases in which any communication, especially those involving social media, has grown to have serious implications for the author [23] and, potentially, the institution they are associated with.

## Rule 6: Tell a story

A good comic, like a good scientific manuscript, tells a story. Like a story, a comic has a beginning (the setup), a middle (the conflict), and a resolution (the punchline). A single-panel comic compresses all these into a single illustration, but it may lay out all the elements of the story in the panel (Fig 6). If illustrating a process or mechanism, start with Rule 4 and personify the elements. Then, think about the story your comic is telling—the steps of the process—and how this might be made more memorable by using your characters. What would the enzyme in your comic say if it could talk? You’ve just given the enzyme that ability! All stories have conflict. This can be in the form of an actual villain, a conflict of ideas, an unseen context to the story, or a joke that the reader is likely to understand. It is important that the language you use to help tell this story be simple and legible. Ideally, it should be tested on nonnative speakers. The impact of the comic can be highly reduced if readers don’t understand the dialogue.

## Rule 7: Draw on what you know and find your own voice

As with many other things, the adage “write what you know” applies to comics as well, but don’t feel limited to only what you’re an expert in. Draw from your own experience (paying attention to Rule 5, of course), and if you are comfortable taking on difficult problems or ideas, then go ahead. Personal stories that come from your own experience and emotions can be incredibly powerful [24]. Your comics might be topical, but that’s ok—science is topical. And by bringing something that you care about and understand to a wider audience, you might just communicate outside your subspecialty. Paying attention to concepts you find important, issues that are relevant to you, and interactions you have daily can be a treasure trove of ideas if you pay attention. If you have a comic or an idea for a comic, try bouncing it off a trusted friend or colleague (Fig 7); then, take their feedback and use it to improve your ideas iteratively. It may take time to find what subjects you like to focus on and how you like to represent ideas, and that’s ok. Art, like science, is a continually evolving process, and it is important to find your own voice.

## Rule 8: Use your imagination

Readers expect comics to be imaginative and to depict ideas in new, fresh ways. A great way to communicate complex or esoteric concepts is to use analogies. Analogies allow the reader to make a connection between something that they can relate to and abstract concepts that may be complex and hard to grasp. An added benefit of analogies is that they often allow for simple variations to make a subject humorous. For example, you can equip a car with multiple “accessories” to depict the process of peer review [18] or transform a dot and circle into an acorn and a squash (Fig 8). However, be careful with analogies because they can sometimes lead to incorrect conclusions about a topic.

## Rule 9: Sketch and draft

One of the most important aspects of an effective comic is clear communication. Storyboard ideas with quick sketches. Lay out the important bits of the comic: where you want the characters, how you want the panels arranged, and where the text will go. This last point, where the text will go, is actually really important and sometimes difficult to do. Experiment with it if it doesn’t seem right the first time. Choose your words. Just like a joke given by a standup comedienne, the difference between a great joke and a dud can sometimes be the specific way that you deliver it and the words that you use. You usually won’t give a talk at a conference off-the-cuff, so don’t do it here either! Test ideas out on others first. Write down a few ideas if you are having trouble. Sometimes the first thing that pops into your head is the best. Other times, an idea needs coaxing and refinement to really shine (Fig 9). You’ll learn to recognize the difference between the two.

## Rule 10: Practice, practice, practice and have fun

No one becomes great at something instantly. Give yourself time and practice often. Sketch at conferences (see [17]), doodle during down time, and carry a notebook for ideas. Learn from others. Read some comics. There are some great ones out there and new ones popping up all the time. Find some that resonate with you and draw inspiration from them. Remember, if you have an idea, you can start without needing to do any drawing at all [19]. Use social media like Twitter, Facebook, Tumblr, and Instagram to reach your audience. Start an account for your comic and it will start to take on a life of its own! Most of all, have fun (Fig 10). Let’s make that a rule.

If you are still reading, take out a piece of paper and draw a circle. Now give it some eyes and a mouth. Now have it thinking or saying something about science. Did it work? Congratulations! You are now a science comic artist!

## References

[1] Concannon C. and Grenon M., Researchers: share your passion for science! Biochem Soc Trans, 2016 44(5): p. 1507–1515. doi: 10.1042/BST20160086 27911733 10.1042/BST20160086

[2] McClain C. and Neeley L., A critical evaluation of science outreach via social media: its role and impact on scientists. F1000Res, 2014 3: p. 300 doi: 10.12688/f1000research.5918.2 25866620 10.12688/f1000research.5918.1 PMC4376169

[3] McClain C.R., Practices and promises of Facebook for science outreach: Becoming a "Nerd of Trust". PLoS Biol, 2017 15(6): p. e2002020 doi: 10.1371/journal.pbio.2002020 28654674 10.1371/journal.pbio.2002020 PMC5486963

[4] Munroe, R. xkcd: A webcomic of romance, sarcasm, math, and language.; Available from: http://xkcd.com/. Accessed on 20 July 2017.

[5] Munroe, R., Earth Temperature Timeline.; Available from: http://xkcd.com/1732/. Accessed on 20 July 2017.

[6] Munroe, R., Old Files.; Available from: https://xkcd.com/1360/. Accessed on 20 July 2017.

[7] Cham, J. PhD: Piled Higher and Deeper. Available from: http://phdcomics.com/. Accessed on 20 July 2017.

[8] Partridge, M. Errant Science. Available from: http://errantscience.com/. Accessed on 20 July 2017.

[9] McDermott, J. RedPen/BlackPen. Available from: http://redpenblackpen.twitter.com/. Accessed on 20 July 2017.

[10] Endy D., Foundations for engineering biology. Nature, 2005 438(7067): p. 449–53. doi: 10.1038/nature04342 16306983 10.1038/nature04342

[11] Briscoe A.D., et al, Female behaviour drives expression and evolution of gustatory receptors in butterflies. PLoS Genet, 2013 9(7): p. e1003620 doi: 10.1371/journal.pgen.1003620 23950722 10.1371/journal.pgen.1003620 PMC3732137

[12] Caudron F. and Barral Y., A super-assembly of Whi3 encodes memory of deceptive encounters by single cells during yeast courtship. Cell, 2013 155(6): p. 1244–57. doi: 10.1016/j.cell.2013.10.046 24315096 10.1016/j.cell.2013.10.046

[13] Krakow M., Graphic Narratives and Cancer Prevention: A Case Study of an American Cancer Society Comic Book. Health Commun, 2017 32(5): p. 525–528. doi: 10.1080/10410236.2016.1211075 27542072 10.1080/10410236.2016.1211075

[14] Tarver T., et al, A Novel tool for Health Literacy: Using Comic Books to Combat Childhood Obesity. J Hosp Librariansh, 2016 16(2): p. 152–159. doi: 10.1080/15323269.2016.1154768 27840597 10.1080/15323269.2016.1154768 PMC5102389

[15] McNicol S., Humanising illness: presenting health information in educational comics. Med Humanit, 2014 40(1): p. 49–55. doi: 10.1136/medhum-2013-010469 24398159 10.1136/medhum-2013-010469

[16] Thébaud O., et al, Managing marine socio-ecological systems: picturing the future. ICES J Mar Sci, 2017 fsw252.

[17] Rohde M, Toselli M, and A. B. Sketchnote Army. Available from: http://sketchnotearmy.com/. Accessed on 20 September 2017.

[18] McDermott J., Your Manuscript on Peer Review. Journal of Vascular and Interventional Radiology 2017 28(5): p. 748.

[19] Kessler, S. 6 Free Sites for Creating Your Own Comics. 2010; Available from: http://mashable.com/2010/10/24/create-your-own-comics/. Accessed on 20 September 2017.

[20] How to Make a Comic Book. Available from: http://www.wikihow.com/Make-a-Comic-Book. Accessed on 20 September 2017.

[21] D'Hont A., et al, The banana (Musa acuminata) genome and the evolution of monocotyledonous plants. Nature, 2012 488(7410): p. 213–7. doi: 10.1038/nature11241 22801500 10.1038/nature11241

[22] Harmon, D., Pilot S01E01, in Community. 2009.

[23] Feldman, B. Talking to the Man Behind ‘Loss,’ the Internet’s Longest-Running Miscarriage ‘Joke’. 2015; Available from: http://nymag.com/selectall/2015/11/longest-running-miscarriage-meme-on-the-web.html. Accessed on 20 September 2017.

[24] Weaver-Hightower M.B., Losing Thomas & Ella: A Father's Story (A Research Comic). J Med Humanit, 2017 38(3): p. 215–230. doi: 10.1007/s10912-015-9359-z 26463352 10.1007/s10912-015-9359-z

